# Scaling of quantitative cardiomyocyte properties in the left ventricle of different mammalian species

**DOI:** 10.1242/jeb.249489

**Published:** 2025-01-10

**Authors:** Tanja Kloock, David J. Jörg, Christian Mühlfeld

**Affiliations:** ^1^ Hannover Medical School, Institute of Functional and Applied Anatomy, 30625 Hanover, Germany; ^2^ Independent Researcher, 60438 Frankfurt am Main, Germany

**Keywords:** Allometry, Cardiomyocyte number, Stereology, Cardiac force, Myofibrils, Mitochondria

## Abstract

Small mammals have a higher heart rate and, relative to body mass (*M*_b_), a higher metabolic rate than large mammals. In contrast, heart weight and stroke volume scale linearly with *M*_b_. With mitochondria filling approximately 50% of a shrew cardiomyocyte – space unavailable for myofibrils – it is unclear how small mammals generate enough contractile force to pump blood into circulation. Here, we investigated whether the total number or volume of cardiomyocytes in the left ventricle compensates for allometry-related volume shifts of cardiac mitochondria and myofibrils. Through statistical analysis of data from 25 studies with 19 different mammalian species with *M*_b_ spanning seven orders of magnitude (2.2 g to 920 kg), we determined how number, volume density and total volume of cardiomyocytes, mitochondria and myofibrils in the left ventricle depend on *M*_b_. We found that these biological variables follow scaling relationships and are proportional to a power *b* of *M*_b_. The number [*b*=1.02 (95% CI: 0.89, 1.14); *t*-test for *b*=1: *P*=0.72] and volume [*b*=0.95 (95% CI: 0.89, 1.03); *t*-test for *b*=1: *P*=0.18] of cardiomyocytes in the left ventricle increases linearly with increasing *M*_b_. In cardiomyocytes, volume density of mitochondria decreases [*b*=–0.056 (95% CI: −0.08, −0.04); *t*-test for *b*=0: *P*<0.0001] and that of myofibrils increases [*b*=0.024 (95%CI: 0.01, 0.04); *t*-test for *b*=0: *P*<0.01] with increasing *M*_b_. Thus, the number or volume of left ventricular cardiomyocytes does not compensate for the higher heart rate and specific metabolic rate of small mammals although a higher mitochondrial and lower myofibrillar volume per cardiomyocyte are present.

## Teaser

**Summary:** Hearts of small mammals have the same number and volume of cardiomyocytes per body mass but a higher mitochondrial and lower myofibrillar volume per cardiomyocyte compared with large mammals.

## INTRODUCTION

The body mass (*M*_b_) of mammals ranges from 2 g in one of the smallest terrestrial mammals, the Etruscan shrew, to several tonnes in the elephant. One hundred and fifty years ago, Max Rubner found a correlation between metabolic rate and body massOne which later became known as Rubner's law of surface metabolism (Rubner, 1883). In the early 20th century, Max Kleiber found that metabolic rate *B* and *M*_b_ are connected through a power-law relationship, *B*∼(*M*_b_)*^b^*, with an exponent of *b*=0.75 (Kleiber, 1932). Following this discovery, many studies were conducted on the relationship between *M*_b_ and various anatomical and physiological variables such as metabolic rate, heart rate, heart weight, volume and volume density of mitochondria and myofibrils. Probably the best known variables that do not scale isometrically with body mass are the weight-specific metabolic rate and oxygen consumption (da Silva et al., 2006; Kleiber, 1932; Lindstedt and Schaeffer, 2002; Rubner, 1883), which decrease with increasing *M*_b_. Such relationships describing body composition or physiological variables that scale with body mass in a disproportionate way have been studied since the 19th century and are called allometric relationships (Huxley and Teissier, 1936; Rubner, 1883; Snell, 1892). Mathematically, allometric relationships are characterized by a power law, *Y*=a*X^b^*, where *Y* denotes the biological variable of interest, *b* denotes the scaling exponent of the independent physiological variable *X*, which is typically body mass and *a* is the variable specific coefficient (Huxley and Teissier, 1936). For three-dimensional variables such as volume or mass, it is called isometry if the variable of interest increases/decreases linearly with body mass, meaning that *b*=1 (Calder, 1987, 1984; Klatt and Vorsteher, 1923; Mandarim-De-Lacerda, 2019; Shingleton, 2010). In contrast, allometric relationships are present if *b* is different from the isotropic value, so the variable of interest increases faster or slower than *M*_b_.

**Fig. 1.**
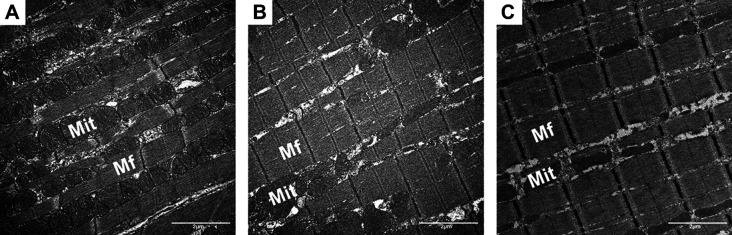
**Electron micrographs of cardiomyocytes.** Cardiomyocytes in (A) Etruscan shrew, (B) dog and (C) horse taken with a Morgagni 268 (FEI, Eindhoven, NL) electron microscope. The volume fraction of mitochondria (Mit) is highest in the Etruscan shrew (A) and lowest in the horse (C) while volume fraction of myofibrils (Mf) is highest in the horse (C) and lowest in the Etruscan shrew (A) (V_v_(Mit/Cm): 36% Etruscan shrew, 23% dog, 19% horse). In contrast, volume fraction of myofibrils shows the opposite relationship (*V*_v_(Mf/Cm): 50% Etruscan shrew, 63% dog, 63% horse).

Heart mass and stroke volume are known to scale isometrically (Günther, 1975; Lindstedt and Schaeffer, 2002). A possible increase in blood pressure across terrestrial mammals with increasing *M*_b_ is discussed controversially within the literature (Günther, 1975; Poulsen et al., 2018; Sandal et al., 2020; Snelling and Seymour, 2024; Seymour and Blaylock, 2000; White and Seymour, 2014). In contrast, cardiac mitochondrial volume (Hoppeler et al., 1984; Snelling et al., 2016), resting heart rate (Holt et al., 1968) and cardiac output (Lindstedt and Schaeffer, 2002) do not scale isometrically with body mass. As such, when related to body mass, smaller mammals have a higher resting heart rate (*M*_b_ ^−0.25^) and a higher left ventricular volume density (*V*_v_) of mitochondria per cardiomyocyte (*M*_b_ ^−0.044^) compared with larger mammals (Fig. 1) (Hoppeler et al., 1984; Horrell et al., 2022; Snelling et al., 2016). The left ventricular *V*_v_ of contractile filaments – the myofibrils – per cardiomyocyte is controversial in the literature: Schipke et al. (2014a) and Snelling et al. (2016) found an allometric relationship (*M*_b_ ^0.022^) whereas this value of *V*_v_ was found to be similar in all species in an earlier study by Barth et al. (1992).

If approximately half of the cardiomyocyte volume is occupied by the organelles that generate the energy for contraction, then how do small mammals manage to provide enough force to pump blood through their body? Since the scaling of left ventricular volume density of myofibrils per cardiomyocyte is disputed, we hypothesized that smaller mammals possess a relatively higher cardiomyocyte number or volume per gram of body mass than larger mammals. To this end, a literature search was performed to collect reliable data on the number, volume density and volume of cardiomyocytes, mitochondria and myofibrils in the left ventricle of different mammalian species with different body mass. Regression analyses were performed to examine the scaling relationship with body mass.

**Abbreviations**

*M*_b_: body mass
Cm: cardiomyocyte
LV: left ventricle
Mf: myofibrils
Mit: mitochondria
*N*: number
*N*(Cm,LV): number of cardiomyocytes in the left ventricle
*V*: volume
*V*(Cm,LV): absolute volume of cardiomyocytes in the left ventricle
*V*(Mf,LV): absolute volume of myofibrils in the left ventricle
*V*(Mit,LV): absolute volume of mitochondria in the left ventricle
*V*_v_: volume density
*V*_v_(Cm/LV): volume density of cardiomyocytes per left ventricle
*V*_v_(Mf/Cm): volume density of myofibrils per cardiomyocyte
*V*_v_(Mit/Cm): volume density of mitochondria per cardiomyocyte

## MATERIALS AND METHODS

The literature was searched for studies on the number (*N*), volume (*V*) and volume density (*V*_v_) of cardiomyocytes, mitochondria and myofibrils in the left ventricle (LV) of various terrestrial mammalian species. Scientific databases (PubMed, Google Scholar) were searched entering different keywords and combinations (cardiomyocyte, number, stereology, volume, allometry, mitochondria, myofibrils). Because very specific data were needed to fit the following inclusion criteria, only a few studies could be found this way. Therefore, a focused search proceeded. Emphasis was placed on species diversity and the greatest possible range of body mass.

### Inclusion criteria

Several methodological and biological factors influence the number of cardiomyocytes. Inclusion criteria were set accordingly (Table 1).

**Table 1.** Inclusion criteria for the literature search based on influencing parameters

Influencing parameter	Inclusion criterion
Allometric formula: *Y*=*aM*_b_*^b^*	Body mass published
Pathological processes: e.g. intrauterine growth restriction, diet	Healthy animals
Left or right ventricle	Left ventricle
Age	Age older than ∼1/10 life expectancy*
Methods: correction for multinucleation, ‘reference trap’	Stereology, cardiomyocyte number corrected for multinucleation^‡^

*For studies about number of cardiomyocytes.

^‡^Owing to lack of human data, inclusion of one study without correction for multinucleation (Tang et al., 2009).

First, many pathological processes have been described that alter cardiomyocyte number such as intrauterine growth restriction (Botting et al., 2012, 2014, 2018; Corstius et al., 2005; Gezmish et al., 2010; Lim et al., 2010; Schipke et al., 2017; Vranas et al., 2017) and diet (Aguila and Alberto Mandarim-de-Lacerda, 2001; Schipke et al., 2014b). Therefore, only data from healthy mammals (mostly control groups) were included. Under physiological conditions, the postnatal left ventricle is composed of more muscle mass than the right ventricle (RV) because it has to generate higher pressures. Nevertheless, the chamber mass ratio of LV:RV is only 3:1, whereas the LV:RV blood pressure ratio is 6:1, which is assumed to be the case owing to a smaller radius of curvature in the left ventricle leading to mechanical advantage in pressure development (Snelling et al., 2015). Therefore, the left ventricle contains a higher number of cardiomyocytes than the right ventricle (Gezmish et al., 2010). Since most studies investigating the number of cardiomyocytes have been performed on the left ventricle only, and there is little data available for the right ventricle, we focused our analysis on the left ventricle. Because the left ventricle pumps the blood into the systemic circulation it has to generate higher pressures to overcome both the resistance of the systemic circuit and the vertical distance between heart and brain which differs with body size.

At which point in life the final number of cardiomyocytes is reached is a controversial topic. In mice, no cardiomyocyte proliferation could be detected after day 15 (Naqvi et al., 2014), whereas in human hearts, proliferation was found to decrease after the first decade of life (Bergmann et al., 2015). In juvenile rats, cardiomyocyte proliferation could be induced by exercise, but not in adult rats (Asif et al., 2018). In addition, Brüel et al. (2007) were able to increase the number of cardiomyocytes by administration of growth hormone in rats. In order to obtain comparable data, only studies were included in which the animals had presumably reached their final number of cardiomyocytes because of cessation of proliferation.

Stereology is a method of analyzing numbers, sizes, volumes and other geometric variables in three-dimensional objects by using two-dimensional sections. It is widely used when variables are measured in flat histological sections and their three-dimensional extent is calculated (Royet, 1991). Design-based stereology is simple, precise and efficient and is therefore considered the gold standard in quantitative microscopy (Mühlfeld et al., 2010). In the literature, various stereological methods are used to count cardiomyocytes, which may lead to different results. In general, the total number of mononucleated cells can be estimated by counting the number of nuclei in a disector (Sterio, 1984). Cardiomyocytes are mono-, bi- or multinucleated cells, with the number of nuclei changing during development or disease. Therefore, direct estimation of cell number by counting of nuclei as a surrogate variable is not possible. After counting the total number of nuclei, a second step is to estimate the mean number of nuclei per cardiomyocyte from serial sections (Brüel et al., 2005). Other methods to estimate the mean number of nuclei per cardiomyocyte include confocal laser scanning microscopy of thick sections after fluorescence staining (Schipke et al., 2014b) and flow cytometry after enzymatic digestion of hearts (Corstius et al., 2005). Once the total and the mean number of nuclei per cardiomyocyte are determined, the total number of cardiomyocytes can be calculated. Unlike stereology, flow cytometry typically does not count nuclei from the same samples used to count other variables. Furthermore, flow cytometry cannot distinguish between multinucleation and polyploidy (Mühlfeld and Schipke, 2022). Therefore, the number of cardiomyocytes determined by stereology is more reliable than that determined by flow cytometry.

Care must be taken to avoid the ‘reference trap’ (Mayhew et al., 2003). The number of cells is often assessed by counting the total number of cells in a given field of view. The use of ratios is considered obsolete because they are prone to error because of changes in either the denominator or the numerator (Cruz-Orive, 2006; Miles and Davy, 1976). Therefore, the number of cells should always be related to a specific reference volume, such as the left ventricle (Mühlfeld and Schipke, 2022). Furthermore, the number of particles is a zero-dimensional variable and cannot be assessed in a two-dimensional section, but requires a three-dimensional test system (Ochs and Mühlfeld, 2013). Because of these methodological issues, only studies that corrected the number of cardiomyocytes for multinucleation and used standard stereological methods were included. However, owing to the lack of human data, one study was included that did not correct for multinucleation in human hearts (Tang et al., 2009).

Considering the above-mentioned factors influencing the number of cardiomyocytes, studies were only included if they used stereology and published data of the left ventricle of healthy, control or sham animals. Studies were excluded if body mass was not reported. Data on the number of cardiomyocytes were only included if the animals were older than 1/10 of life expectancy, as the number of cardiomyocytes is expected to remain constant from this age on or even earlier (Bergmann et al., 2015; Naqvi et al., 2014).

As the stereological methods to estimate the number of objects became available only within the last 40 years (Brüel and Nyengaard, 2005; Sterio, 1984), there is a lot more data about volume densities than numbers published. Those studies were included when they used standard stereological methods such as the point-counting method, where the number of points hitting a certain reference volume is counted and volumes as well as volume densities are estimated (Chalkley, 1943; Mühlfeld et al., 2010).

### Collected data

Datasets of different species were collected on body mass (*M*_b_), volume density [*V*_v_(Cm/LV)], total volume [*V*(Cm,LV)], and number of cardiomyocytes in the left ventricle [*N*(Cm,LV)], as well as volume density per cardiomyocyte and absolute volume of mitochondria [*V*_v_(Mit/Cm), *V*(Mit,LV)] and myofibrils [*V*_v_(Mf/Cm), (*V*Mf,LV)] in the left ventricle (Table S1). The ratio *V*_v_(Mit/Cm)/*V*_v_(Mf/Cm) was calculated. Units were standardised and averages were calculated when multiple values were reported (e.g. from different sites or animals) or when ranges were published (e.g. body mass). Species means were calculated whenever there was more than one dataset per species, even though there was not a great difference between analysis performed across individuals compared to species means (Tables S2 and S3).

### Statistics

Allometric scaling relationships of the form *Y*=*a* · *X^b^* between two physiological quantities *Y* and *X*, where *a* is a constant prefactor and *b* is the allometric scaling exponent, were determined by a regression analysis of log-transformed data. Three different regression methods (linear regression, major axis regression and standardised major axis regression) (Warton et al., 2006) were applied and compared to confirm independence of the results on the choice of the regression method. Species means were calculated.

Linear regression was chosen as the reference method reported in the main text, since it is widely used and the better comparable method. Nevertheless, calculated exponents were similar across all three methods (Tables S2 and S3).

Regression results are reported as regression slope and 95% confidence interval. *t*-tests were performed to test for statistical significance of the differences of the regression slopes from 1 (i.e. from isometry for volumes, masses and numbers) or from 0 (i.e. from constancy for volume densities); the corresponding *P*-values are denoted by *P*_1_ and *P*_0_, respectively.

Data analysis was performed using an implementation of the different regression methods and statistical tests in Python 3.11 using NumPy 1.24.3.

As this study is a meta-analysis incorporating many different studies from different experimenters using different methods, methodical inaccuracy is expected. Therefore, the impact of phylogenetic relation is believed to be rather small, which is why no correction for phylogenetic relationships was made.

## RESULTS

**Fig. 2.**
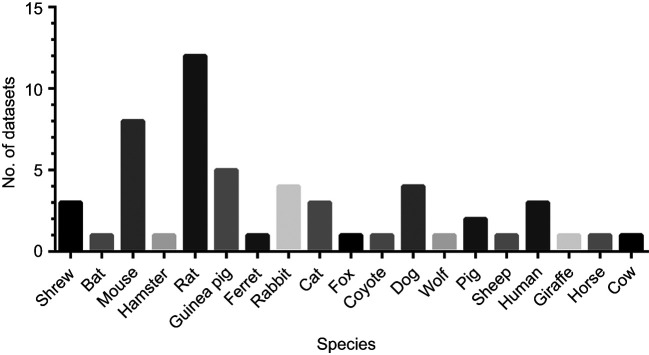
Number of datasets for the species included in this study.

A total of 25 studies met the criteria and were included in the present study, resulting in 54 different datasets (range: 1–12 datasets, median: 1 dataset) (Table 2). Datasets were from 19 different species (shrew, bat, mouse, hamster, rat, guinea pig, ferret, rabbit, cat, fox, coyote, dog, wolf, pig, sheep, human, giraffe, horse, cow) with a range of body masses from 2.2 g to 920 kg (Fig. 2). For most of the datasets, sex was not provided. Eight datasets were from female animals, 15 datasets were from male animals and 7 datasets were a mix from both sexes.

**Table 2.** Studies and species included in the analysis

Study	Variable	Species
Anversa et al., 1971	*V*_v_(Mit/Cm), *V*_v_(Mf/Cm), *V*_v_(Mit/Cm)/*V*_v_(Mf/Cm)	Rabbit
Appell et al., 1983	*V*_v_(Mit/Cm), *V*_v_(Mf/Cm), *V*_v_(Mit/Cm)/*V*_v_(Mf/Cm)	Guinea pig
Asif et al., 2018	*N*(Cm,LV)	Rat
Barth et al., 1992	*V*_v_(Mit/Cm), *V*_v_(Mf/Cm), *V*_v_(Mit/Cm)/*V*_v_(Mf/Cm)	Bat, mouse, rat, guinea pig, ferret, rabbit, cat, dog, pig, human
Botting et al., 2018	*N*(Cm,LV)	Guinea pig
Breisch, 1982	*V*_v_(Mit/Cm), *V*_v_(Mf/Cm), *V*_v_(Mit/Cm)/*V*_v_(Mf/Cm)	Cat
Brüel et al., 2007	*N*(Cm,LV), *V*_v_(Cm/LV), *V*(Cm,LV)	Rat
Crisman and Tomanek, 1985	*V*_v_(Mit/Cm), *V*_v_(Mf/Cm), *V*_v_(Mit/Cm)/*V*_v_(Mf/Cm)	Rat
Eisele et al., 2008	*N*(Cm,LV), *V*_v_(Cm/LV), *V*(Cm,LV), *V*(Mit,LV), *V*_v_(Mit/LV)	Mouse
Frenzel et al., 1988	*V*_v_(Mit/Cm), *V*_v_(Mf/Cm), *V*_v_(Mit/Cm)/*V*_v_(Mf/Cm)	Rat
Goldstein et al., 1974	*V*_v_(Mit/Cm), *V*_v_(Mf/Cm), *V*_v_(Mit/Cm)/*V*_v_(Mf/Cm)	Rabbit
Hatt et al., 1978	*V*_v_(Mit/Cm), *V*_v_(Mf/Cm), *V*_v_(Mit/Cm)/*V*_v_(Mf/Cm)	Rat
Kim et al., 1994	*V*_v_(Mit/Cm), *V*_v_(Mf/Cm), *V*_v_(Mit/Cm)/*V*_v_(Mf/Cm)	Rat, guinea pig, rabbit
McCallister et al., 1978	*V*_v_(Mit/Cm), *V*_v_(Mf/Cm), *V*_v_(Mit/Cm)/*V*_v_(Mf/Cm)	Dog
Oron and Mandelberg, 1985	*V*_v_(Mit/Cm)	Shrew, mouse, rat, mouse
Østergaard et al., 2013	*N*(Cm,LV), *V*_v_(Cm/LV), *V*(Cm,LV)	Giraffe
Page et al., 1971	*V*_v_(Mit/Cm), *V*_v_(Mf/Cm), *V*_v_(Mit/Cm)/*V*_v_(Mf/Cm)	Rat
Page et al., 1972	*V*_v_(Mit/Cm), *V*_v_(Mf/Cm), *V*_v_(Mit/Cm)/*V*_v_(Mf/Cm)	Rat
Schaper et al., 1985	*V*_v_(Mit/Cm), *V*_v_(Mf/Cm), *V*_v_(Mit/Cm)/*V*_v_(Mf/Cm)	Mouse, hamster, rat, dog, human
Schipke et al., 2014a	*V*_v_(Cm/LV), *V*(Cm,LV), *V*_v_(Mit/Cm), *V*_v_(Mf/Cm), *V*(Mit,LV), *V*(Mf,LV), *V*_v_(Mit/Cm)/*V*_v_(Mf/Cm)	Shrew, mouse, rat, cat, fox, coyote, dog, wolf, horse, cow
Schipke et al., 2014b	*N*(Cm,LV), *V*_v_(Cm/LV), *V*(Cm,LV), *V*_v_(Mf/Cm), *V*(Mit,LV), *V*(Mf,LV), *V*_v_(Mit/Cm)/*V*_v_(Mf/Cm)	Mouse
Schipke et al., 2016	*N*(Cm,LV)	Mouse
Singh et al., 1981	V_v_(Mit/Cm), *V*_v_(Mf/Cm), *V*_v_(Mit/Cm)/*V*_v_(Mf/Cm)	Pig
Tang et al., 2009	*N*(Cm,LV), *V*_v_(Cm/LV), *V*(Cm,LV)	Human
Vranas et al., 2017	*N*(Cm,LV)	Sheep

### Cardiomyocytes

**Fig. 3.**
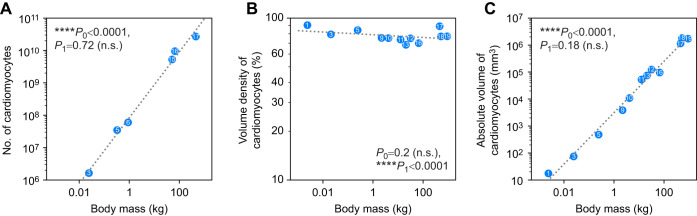
**Allometric relationships of number, volume density and absolute volume of cardiomyocytes in the left ventricle presented as log–log plots.** (A) Number of cardiomyocytes [*N*(Cm,LV)] (*n*=6). (B) Volume density of cardiomyocytes in the left ventricle [*V*_v_(Cm/LV)] (*n*=12). (C) Absolute volume of cardiomyocytes in the left ventricle [*V*(Cm,LV)] (*n*=12). The scaling exponent *b* (95% CI) and the constant *a* are shown in Table 3. Significant differences to a slope of 0 (*P*_0_) and 1 (*P*_1_) (n.s., not significant) are indicated. Included species are presented with numbers: 1=shrew, 2=bat, 3=mouse, 4=hamster, 5=rat, 6=guinea pig, 7=ferret, 8=rabbit, 9=cat, 10=fox, 11=coyote, 12=dog, 13=wolf, 14=pig, 15=sheep, 16=human, 17=giraffe, 18=horse, 19=cow.

The number [*N*(Cm,LV)] and volume [*V*(Cm,LV)] of cardiomyocytes in the left ventricle increased linearly with body mass (i.e. isometrically). Their scaling exponents were determined as 1.02 (95% CI: 0.89, 1.14) (*P*_1_=0.72) and 0.95 (95% CI: 0.88, 1.03) (*P*_1_=0.18), respectively. The volume density *V*_v_(Cm/LV) was not significantly associated with body mass, with an exponent of −0.008 (95% CI: −0.022, 0.005) (*P*_0_=0.20) (Fig. 3, Table 3).

**Table 3.** Slopes for analysed variables of species means and their significance (*Y*=a*X*^b^)

Variable	Scaling exponent *b* (95% CI)	Constant *a* (95% CI)	Significance
*N*(Cm,LV)	1.02 (0.89, 1.14)	8.35×10^7^ (5.60×10^7^, 1.25×10^8^)	n.s. (*P*_1_)
*V*_v_(Cm/LV) (%)	−0.008 (−0.022, 0.005)	0.79 (0.75, 0.83)	n.s. (*P*_0_)
*V*(Cm,LV) (cm³)	0.95 (0.89, 1.03)	3110 (2320, 4170)	n.s. (*P*_1_)
*V*_v_(Mit/Cm) (%)	−0.056 (−0.076, −0.035)	27.3 (25.5, 29.2)	*P*_0_<0.0001
*V*(Mit,LV) (cm³)	0.89 (0.81, 0.96)	0.87 (0.65, 1.15)	*P*_1_<0.01
*V*_v_(Mf/Cm) (%)	0.024 (0.01, 0.04)	59.2 (56.6, 61.9)	*P*_0_<0.01
*V*(Mf,LV) (cm³)	0.99 (0.90, 1.08)	1.97 (1.40, 2.76)	n.s. (*P*_1_)
*V*_v_(Mit/Cm)/*V*_v_(Mf/Cm)	−0.085 (−0.12, −0.056)	47.0 (42.5, 52.0)	*P*_0_<0.0001

*N*, number; *V*_v_, volume density; *V*, total volume; Cm, cardiomyocyte; LV, left ventricle; Mit, mitochondria; Mf, myofibril.

n.s., not significant; *P*_0_, significant difference to *b*=0; *P*_1_, significant difference to *b*=1.

### Mitochondria

**Fig. 4.**
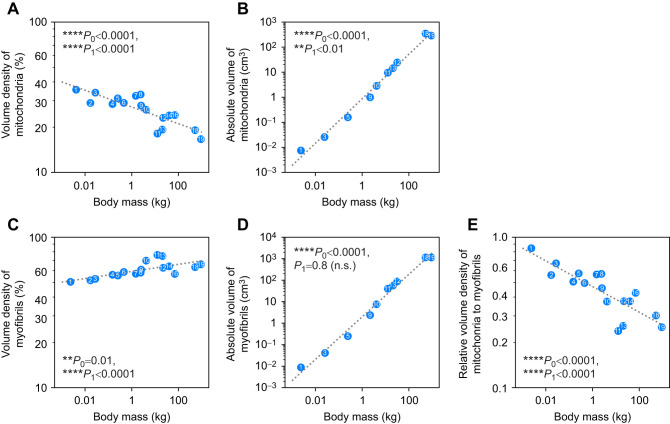
**Allometric relationships of volume density and absolute volume of mitochondria and myofibrils in the left ventricle presented as log–log plots.** (A) Volume density [*V*_v_(Mit/Cm)] (*n*=17) and (B) absolute volume [*V*(Mit,LV)] (*n*=10) of mitochondria in the left ventricle. (C) Volume density [*V*_v_(Mf/Cm)] (*n*=17) and (D) absolute volume [*V*(Mf,LV)] (*n*=10) of myofibrils in the left ventricle. (E) Ratio of *V*_v_(Mit/Cm)/*V*_v_(Mf/Cm) (*n*=17). The scaling exponent *b* (95% CI) and the constant *a* are shown in Table 3. Significant differences to a slope of 0 (*P*_0_) and 1 (*P*_1_) (n.s., not significant) are indicated. Included species are presented with numbers: 1=shrew, 2=bat, 3=mouse, 4=hamster, 5=rat, 6=guinea pig, 7=ferret, 8=rabbit, 9=cat, 10=fox, 11=coyote, 12=dog, 13=wolf, 14=pig, 15=sheep, 16=human, 17=giraffe, 18=horse, 19=cow.

The volume fraction of mitochondria per cardiomyocyte [*V*_v_(Mit/Cm)] decreased with body mass raised to an exponent of −0.056 (95% CI: −0.08, −0.04) (*P*_0_<0.0001) (Fig. 4A, Table 3), such that small mammals have a larger fraction of cell volume occupied by mitochondria than do large mammals. The absolute volume *V*(Mit,LV) increased more slowly than body mass, with an exponent of 0.89 (95% CI: 0.81, 0.96) (*P*_1_<0.01), meaning that smaller animals have a higher total volume of mitochondria per unit body mass (Fig. 4B, Table 3).

### Myofibrils

The volume density of myofibrils (*V*_v_(Mf/Cm)) increased with body mass with an exponent of 0.024 (95% CI: 0.01, 0.04) (*P*_0_<0.01), meaning that *V*_v_(Mf/Cm) was slightly higher in large mammals (Fig. 4C, Table 3). The total volume *V*(Mf,LV) showed a linear increase with body mass, with an exponent of 0.99 (95% CI: 0.90, 1.08) (*P*_1_=0.8) (Fig. 4D, Table 3). The ratio *V*_v_(Mit/Cm)/*V*_v_(Mf/Cm) decreased with body mass with an exponent of −0.085 (95% CI: −0.12, −0.06) (*P*_0_<0.0001) (Fig. 4E, Table 3), meaning that small mammals have a higher ratio than large ones.

## DISCUSSION

### Cardiomyocytes

The research question of this manuscript was how small mammals manage to provide enough force to pump their blood into the circulation if there is a lower *V*_v_(Mf/Cm). Different metabolic needs between mammalian species with different body mass had no effect on the number or volume of left ventricular cardiomyocytes as both variables increase linearly with *M*_b_.

One way to increase cardiac force without increasing myofibrillar density per cardiomyocyte would be an increase in the number or mean volume of cardiomyocytes. Both would lead to an increase in *V*(Cm,LV) as it is the product of number and mean volume of cardiomyocytes. Results of this study show that there is no allometric relationship between *M*_b_ and the number or total volume of cardiomyocytes when comparing different terrestrial mammalian species. Instead, the number and total volume of cardiomyocytes both show a linear relationship with *M*_b_. A possible explanation could be that the number of contractile filaments is not the limiting factor for contractility, but the mitochondria and therefore the oxygen consumption are. Snelling et al. (2016) proposed a reserve capacity for myofibrils generating tension whereas mitochondria are working close to their limit during heavy exercise.

### Mitochondria

If not by having a greater number or volume of cardiomyocytes compared with large mammals, how do small mammals generate enough force to pump blood into the circulation if that much space is occupied already by mitochondria?

First, our results of a decrease in *V*_v_(Mit/Cm) with increasing *M*_b_ (Barth et al., 1992; Hoppeler et al., 1984; Horrell et al., 2022; Schaper et al., 1985; Schipke et al., 2014a) as well as an increase in *V*(Mit,LV) with increasing *M*_b_ that is slower than the increase in *M*_b_ (Hoppeler et al., 1984; Schipke et al., 2014a) are consistent with the literature. In addition to a higher number of cardiac mitochondria per cardiomyocyte and *M*_b_, small mammals are also known to have a higher metabolic rate and oxygen consumption related to *M*_b_ (Lindstedt and Schaeffer, 2002), as well as a higher heart rate (Günther, 1975). All these factors are correlated (Barth et al., 1992; Schaper et al., 1985). A higher heart rate means more cardiomyocyte contractions per second, resulting in a higher energy demand. This is provided by a higher cardiac mitochondrial mass, which requires a higher cardiac oxygen consumption per *M*_b_. In addition, better vascularization and a higher heart rate are necessary to maintain a higher metabolic rate. Therefore, a greater number of cardiac mitochondria and a higher capillary density are plausible in small mammals as mitochondria provide the energy to pump the blood through the circulation (Horrell et al., 2022).

### Myofibrils

In contrast to mitochondria, small mammals have fewer myofibrils per cardiomyocyte than large mammals. Looking at the literature, Schaper et al. (1985) and Schipke et al. (2014a) also describe *V*_v_(Mf/Cm) to scale to *M*_b_ with an exponent of *b*=0.022. In contrast, Barth et al. (1992) found no species variability from bats to humans and *V*_v_(Mf/Cm) was around 60% in all species, although they did not calculate a scaling exponent. However, *V*_v_(Mf/Cm) in the two smallest species included in that study (mouse and bat) was about 52%, hinting at a possible body mass dependence.

Dobson and Himmelreich (2002) proposed a constant ratio of 2:1 for *V*(Mit,LV) and *V*(Mf,LV), which is independent of *M*_b_. This is in contrast to the present study and many (above mentioned) studies that publish an allometric relationship at least for *V*(Mit,LV). This contradicts a constant ratio for all mammals if the numerator varies with *M*_b_. One reason could be the very small range of *M*_b_ of the species included in this particular study (mice, rats and guinea pigs).

Looking at the myofibrils, what would be the benefit of having more myofibrils in large mammals, or better yet, why would larger mammals need more myofibrils than small mammals?

Laplace's law of surface tension describes the relationship between wall stress, pressure, wall thickness, and radius of spheres. It has been applied to the heart and used to estimate ventricular wall stress for over 100 years (Woods, 1892). Although there are several ways to predict ventricular wall stress in the literature, Laplace's law is quite simple: *K=Pr/*2*d* (*K*=ventricular wall stress, *P*=(blood) pressure, *r*=radius, *d*=wall thickness).

As shown in the present study the total volume of cardiomyocytes in the left ventricle (*V*Cm,LV) increases isometrically with *M*_b_, which suggests that left ventricular volume also scales isometrically with *M*_b_ (endsystolic, endiastolic and stroke volume are known to scale isometrically; Günther, 1975). If volume of cardiomyocytes and volume of LV increase isometrically, then wall thickness and radius would scale with the same proportion to *M*_b_. The ratio radius/wall thickness would therefore be independent of *M*_b_. Seymour and Blaylock (2000) also showed that both radius and wall thickness increase allometrically with the same slope (*b*=0.35), and therefore the ratio of *r/d* is independent of *M*_b_. This study confirms that *V*_v_(Mf/LV) increases in larger mammals with an exponent of 0.02 to *M*_b_. If the functional properties of actin and myosin are the same for all mammals, it would suggest that wall stress would also scale with the same exponent as V_v_(Mf/LV). This assumption is also supported by Seymour and Blaylock (2000), who found an exponent of 0.05 to *M*_b_ for wall stress. Therefore, if the ratio of radius/wall thickness is independent of *M*_b_ and wall stress increases allometrically, we would expect an increase in blood pressure following Laplace's law of surface tension (see above). Seymour and Blaylock (2000) also found a slight increase in blood pressure (*b*=0.05) and wall stress (*b*=0.04) with increasing *M*_b_. A slight increase in blood pressure could explain the increase in myofibrils found in this study with a similar scaling exponent of *b*=0.024. One explanation for an increase in blood pressure with increasing *M*_b_, would be the increasing vertical distance between heart and brain, that needs to be overcome by the oxygenized arterial blood. On the other hand, blood pressure has to be high enough to overcome gravity as well as the total peripheral resistance, which decreases with increasing body mass (Günther, 1975). Overall, the influence of gravity on the heart work and a possible scaling of blood pressure with *M*_b_ is controversially discussed (Günther, 1975; Poulsen et al., 2018; Sandal et al., 2020; Snelling and Seymour, 2024; Seymour and Blaylock, 2000; White and Seymour, 2014). There are different values for blood pressure in large mammals in the literature. In contrast to humans, it is difficult to measure blood pressure in awake large animals. Non-invasive measurement with a cuff is possible, but often gives unreliable results because even small movements of the animal lead to false values. Invasive intra-arterial measurement is also difficult to perform in awake animals. In addition, many variables influence the blood pressure such as anaesthesia, fixation and habituation in animals while measuring blood pressure (Poulsen et al., 2018). It is therefore possible that in large mammals there is indeed a slight increase in blood pressure, which would lead to an increase in wall stress and require a higher number of myofibrils.

When analysing the relationships between ultrastructural composition of cardiomyocytes and their physiological function, it needs to be considered that the maximum function is represented by anatomical properties, as there needs to be a reserve capacity to switch from resting conditions to maximum activity, which is especially important for prey and flight animals. In addition to the reserve capacity, there is a safety margin for many physiological variables as well (Alexander and Wiggers, 1953; Hicks and Wang, 2021; Meltzer, 1907). The reserve capacity for metabolism as well as cardiac output in large mammals is higher than in small mammals (Baudinette, 1978). Nevertheless, the basal (*b*=0.75) and maximum (*b*=0.79) metabolic rates are higher in small mammals when related to *M*_b_ (Calder, 1984; Kleiber, 1932; Taylor et al., 1981). Therefore, this does not change the above presented interpretation of scaling relationships, and small mammals would still be expected to need a higher *V*_v_(Mit/Cm). For myofibrils, it is similar, as larger animals need to create a possibly higher blood pressure during rest as well as during exercise as vertical distance between the heart and brain, as well as gravity, do not change during exercise. Additionally, it would be very interesting to compare athletic and sedentary species in the present study as they have different physiological needs and conditions, which require an adaptation in cardiac ultrastructure (Weibel et al., 1991). Owing to lack of data from those specific pairs of species, this could not be done in our study but is worth acknowledging in future studies.

### Limitations

A limitation of the present study is the lack of standardized methods, as studies performed by different authors were reviewed and analysed retrospectively. In particular, the correction for cardiomyocyte multinucleation was performed in different ways. In addition, more small mammals (mainly mice and rats) were included than large mammals due to lack of data. There was also an uneven distribution of sex of the included datasets, which could lead to potential bias. Because the left ventricle is responsible for the systemic circulation, we only included data from the left ventricle. However, there are studies showing no difference in the electron microscopic composition of cardiomyocytes from the left and the right ventricles (Singh et al., 1981). As explained earlier, there are more available data on volume densities than total numbers of objects because of methodological constraints. Therefore, varying data were used for analysis of the different variables. A prospective stereological study with standardized methods and an equal distribution of species and sex would be necessary to further validate the allometric scaling of cardiomyocytes. In addition, an analysis of the three-dimensional cardiomyocyte size and *in vitro* force measurement would be helpful.

### Conclusions

There is a linear isometric increase in the total number and volume of cardiomyocytes with body mass. Cardiomyocytes of small mammals do have a higher volume density of mitochondria and lower volume density of myofibrils compared with cardiomyocytes of large mammals. Therefore, the myofibrils do not seem to be a limiting factor and provide enough force to pump the blood into the circulation even in small mammals where approximately half of the cell volume is packed with mitochondria.

## Supplementary Material

10.1242/jexbio.249489_sup1 Supplementary information
