# Supplementary material for: Scaling of quantitative cardiomyocyte properties in the left ventricle of different mammalian species
Source: J Exp Biol. 2025 Jan 10;228(1):JEB249489. doi: 10.1242/jeb.249489 (PMC11744323; doi:10.1242/jeb.249489)
Supplement: Supplementary information [file jexbio-228-249489-s1.pdf]

**Table S1.** Number of data sets per species contributing to analysis of different variables.

| Species/ Variable | N(Cm,LV) | $V_v$(Cm/LV) [%] | V(Cm,LV) [cm³] | $V_v$(Mit/Cm) [%] | V(Mit,LV) [cm³] | $V_v$(Mf/Cm) [%] | V(Mf,LV) [cm³] | $V_v$(Mit/Cm)/ $V_v$(Mf/Cm) |
|---|---|---|---|---|---|---|---|---|
| **Shrew** | | 1 | 1 | 3 | 1 | 1 | 1 | 1 |
| **Bat** | | | | 1 | | 1 | | 1 |
| **Mouse** | 4 | 2 | 4 | 7 | 4 | 5 | 3 | 5 |
| **Hamster** | | | | 1 | | 1 | | 1 |
| **Rat** | 2 | 2 | 2 | 10 | 1 | 9 | 1 | 9 |
| **Guinea pig** | 2 | | | 3 | | 3 | | 3 |
| **Ferret** | | | | 1 | | 1 | | 1 |
| **Rabbit** | | | | 4 | | 4 | | 4 |
| **Cat** | | 1 | 1 | 3 | 1 | 3 | 1 | 3 |
| **Fox** | | 1 | 1 | 1 | 1 | 1 | 1 | 1 |
| **Coyote** | | 1 | 1 | 1 | 1 | 1 | 1 | 1 |
| **Dog** | | 1 | 1 | 4 | 1 | 4 | 1 | 4 |
| **Wolf** | | 1 | 1 | 1 | 1 | 1 | 1 | 1 |
| **Pig** | | | | 2 | | 2 | | 2 |
| **Sheep** | 1 | | | | | | | |
| **Human** | 1 | 1 | 1 | 2 | | 2 | | 2 |
| **Giraffe** | 1 | 1 | 1 | | | | | |
| **Horse** | | 1 | 1 | 1 | 1 | 1 | 1 | 1 |
| **Cattle** | | 1 | 1 | 1 | 1 | 1 | 1 | 1 |

**Table S2.** Detailed statistics for species means and comparison of different statistical methods: Linear Regression (LR), Standardised Major Axis (SMA), Major Axis (MA). Calculation was performed with species means.

| **Variable** | **LR** [scaling exponent b (95% CI, significance)] | **MA** [scaling exponent b (95% CI, significance)] | **SMA** [scaling exponent b (95% CI, significance)] |
|---|---|---|---|
| **N(Cm,LV)** | 1.02 (0.89,1.14, $p_1$=0.72) | 1.02 (0.9,1.16, $p_1$=0.66) | 1.02 (0.9,1.15, $p_1$=0.66) |
| **$V_v$(Cm/LV) [%]** | -0.008 (-0.022,0.005, $p_0$=0.2) | -0.008 (-0.02,0.005, $p_0$=0.2) | -0.02 (-0.01,-0.04, $p_0$<0.01) |
| **V(Cm,LV) [cm³]** | 0.95 (0.88,1.03, $p_1$=0.18) | 0.96 (0.89,1.03, $p_1$=0.24) | 0.96 (0.889,1.03, $p_1$=0.24) |
| **$V_v$(Mit/Cm)[%]** | -0.056 (-0.08,-0.04, $p_0$<0.0001) | -0.056 (-0.08,-0.04, $p_0$<0.0001) | -0.067 (-0.08,-0.04, $p_0$<0.0001) |
| **V(Mit,LV) [cm³]** | 0.89 (0.81,0.96, $p_1$<0.01) | 0.89 (0.82,0.97, $p_1$<0.05) | 0.89 (0.82,0.97, $p_1$<0.05) |
| **$V_v$(Mf/Cm) [%]** | 0.024 (0.01,0.04,$p_0$<0.01) | 0.024 (0.01,0.04,$p_0$<0.01) | 0.034 (0.02,0.05,$p_0$<0.0001) |
| **V(Mf,LV) [cm³]** | 0.99 (0.9,1.08, $p_1$=0.8) | 1.0 (0.9,1.09, $p_1$=0.9) | 1.0 (0.9,1.09, $p_1$=0.9) |
| **$V_v$(Mit/Cm)/$V_v$(Mf/Cm)** | -0.085 (-0.12,-0.06, $p_0$<0.0001) | -0.085 (-0.12,-0.06, $p_0$<0.0001) | -0.1 (-0.08,-0.14, $p_0$<0.0001) |

N, number; $V_v$, volume density; V, total volume; Cm, cardiomyocyte; LV, left ventricle; Mit, Mitochondria; Mf, Myofibrils

ns: no significance, *: p<0.05, **: p<0.01, ***: p<0.001, ****: p<0.0001

$p_0$: significant difference to b=0

$p_1$: significant difference to b=1

Journal of Experimental Biology • Supplementary information

**Table S3.** Detailed statistics without species means and comparison of different statistical methods: Linear Regression (LR), Standardised Major Axis (SMA), Major Axis (MA). Calculation was performed without species means.

| Variable | LR [scaling exponent b (95% CI, significance)] | MA [scaling exponent b (95% CI, significance)] | SMA [scaling exponent b (95% CI, significance)] |
|---|---|---|---|
| **N(Cm,LV)** | 1.04 (0.95,1.13, $p_1$=0.37) | 1.04 (1.0,1.14, $p_1$=0.29) | 1.04 (1.0,1.14, $p_1$=0.29) |
| **$V_v$(Cm/LV) [%]** | -0.008 (-0.022,0.005, $p_0$=0.2) | -0.008 (-0.02,0.005, $p_0$=0.2) | -0.02 (-0.01,-0.04, $p_0$<0.01) |
| **V(Cm,LV) [$cm^3$]** | 0.97 (0.91,1.02, $p_1$=0.19) | 0.97 (0.92,1.03, $p_1$=0.26) | 0.97 (0.92,1.03, $p_1$=0.26) |
| **$V_v$(Mit/Cm)[%]** | -0.052 (-0.07,-0.04, $p_0$<0.0001) | -0.053 (-0.07,-0.04, $p_0$<0.0001) | -0.068 (-0.06,-0.08, $p_0$<0.0001) |
| **V(Mit,LV) [$cm^3$]** | 0.9 (0.85,0.95, $p_1$<0.01) | 0.9 (0.85,0.96, $p_1$<0.01) | 0.9 (0.85,0.96, $p_1$<0.01) |
| **$V_v$(Mf/Cm) [%]** | 0.024 (0.01,0.03, $p_0$<0.0001) | 0.024 (0.02,0.03, $p_0$<0.0001) | 0.037 (0.03,0.05, $p_0$<0.0001) |
| **V(Mf,LV) [$cm^3$]** | 0.99 (0.9,1.08, $p_1$=0.8) | 1.0 (0.9,1.09, $p_1$=0.9) | 1.0 (0.9,1.09, $p_1$=0.9) |
| **$V_v$(Mit/Cm)/$V_v$(Mf/Cm)** | -0.085 (-0.11,-0.06, $p_0$<0.0001) | -0.085 (-0.11,-0.06, $p_0$<0.0001) | -0.1 (-0.09,-0.13, $p_0$<0.0001) |

N, number; $V_v$, volume density; V, total volume; Cm, cardiomyocyte; LV, left ventricle; Mit, Mitochondria; Mf, Myofibrils

ns: no significance, *: p<0.05, **: p<0.01, ***: p<0.001, ****: p<0.0001

$p_0$: significant difference to b=0

$p_1$: significant difference to b=1
